# Understanding the genetic basis of the incompatibility of IncK1 and IncK2 plasmids

**DOI:** 10.12688/openreseurope.15121.1

**Published:** 2023-04-11

**Authors:** Marta Rozwandowicz, Arie Kant, Jaap Wagenaar, Dik Mevius, Joost Hordijk, Michael Brouwer

**Affiliations:** 1 National Institute for Public Health and the Environment, Bilthoven, 3721 MA, The Netherlands; 2 Wageningen Bioveterinary Research, Lelystd, 8221 RA, The Netherlands; 3 Utrecht University, Utrecht, 3584 CS, The Netherlands

**Keywords:** plasmid, incompatibility, IncK, minireplicon

## Abstract

Antimicrobial resistance is a persistent challenge in human and veterinary medicine, which is often encoded on plasmids which are transmissible between bacterial cells. Incompatibility is the inability of two plasmids to be stably maintained in one cell which is caused by the presence of identical or closely related shared determinants between two plasmids originating from partition or replication mechanisms. For I-complex plasmids in *Enterobacteriacae*, replication- based incompatibility is caused by the small antisense RNA stem-loop structure called RNAI. The I-complex plasmid group IncK consists of two compatible subgroups, IncK1 and IncK2, for which the RNAI differs only by five nucleotides. In this study we focused on the interaction of the IncK1 and IncK2 RNAI structures by constructing minireplicons containing the replication region of IncK1 or IncK2 plasmids coupled with a kanamycin resistance marker. Using minireplicons excludes involvement of incompatibility mechanisms other than RNAI. Additionally, we performed single nucleotide mutagenesis targeting the five nucleotides that differ between the IncK1 and IncK2 RNAI sequences of these minireplicons. The obtained results show that a single nucleotide change in the RNAI structure is responsible for the compatible phenotype of IncK1 with IncK2 plasmids. Only nucleotides in the RNAI top loop and interior loop have an effect on minireplicon incompatibility with wild type plasmids, while mutations in the stem of the RNAI structure had no significant effect on incompatibility. Understanding the molecular basis of incompatibility is relevant for future *in silico* predictions of plasmid incompatibility.

## Introduction

Antimicrobial resistance is a health threat that is emerging globally and threatens human and veterinary medicine. One of the factors that has facilitated this spread is the transfer of mobile genetic elements between bacteria, for which plasmids are mostly responsible in Gram negative bacteria such as *Escherichia coli* ^1^. In *Enterobacteriaceae* there are 40 plasmid types described, mostly based on their incompatibility ^1, 2^.

Incompatibility is the inability of two plasmids to be stably maintained in one cell. This phenotypic trait was long used as a plasmid typing tool for epidemiological studies but this has now mostly been replaced by molecular diagnostic tools ^3^. Incompatibility is caused by the presence of identical or closely related shared determinants between two plasmids, originating from partition or replication mechanisms ^4^.

Replication and the copy number of plasmids can be regulated by antisense RNA. For IncF, IncL, IncM and I-complex plasmids (containing IncI, IncK, IncB/O and IncZ plasmids), incompatibility is mediated by an antisense stem-loop RNA structure (RNAI) that inhibits translation of the *rep* mRNA ^5– 11^.

Involvement of the antisense RNAI in replication control and incompatibility, was extensively studied for I-complex plasmids ^12– 17^. RNAI controls replication through interaction with stem-loop I (SLI) ^18, 19^. The most important step is the formation of the stable kissing complex by pairing between the single-stranded RNAI and SLI ^19^.

Minireplicons can be used to determine if incompatibility of IncK plasmids is determined by the replication control region, and not the partitioning region. Minireplicons contain a minimal part of a plasmid that can replicate at the same copy number as the original plasmid and that maintains the same incompatibility behaviour of the original plasmid ^20^. The role of nucleotides in the top loop domain and upper stem of the RNAI structure in the interaction with the stem-loop I (SLI) was studied in detail for IncB/O plasmids ^12^. IncB/O and IncK plasmids share a high degree of similarity in their RNAI structures. Using systematic mutagenesis of nucleotides in the RNAI structure, it was concluded that for IncB/O plasmids, three nucleotides on the top of the top loop (C37, C38 and C39) are crucial for the initial kissing interaction of RNAI with SLI. The interior loop in the upper stem is involved in the intra-strand melting and inter-strand pairing of RNAI with SLI ^21^. Mutations disrupting the structure of the interior loop have a significant effect on plasmid compatibility ^12^. Mutations at other positions only had significant effects if the mutation caused a base mismatch and therefore altered the structure. 

Two compatible lineages of IncK plasmids were described in literature ^22, 23^. Four SNPs and one indel were identified that differ in the RNAI sequence of IncK1 and IncK2 plasmids, which may contribute to the compatibility and copy number of these plasmids. RNAI is a target allowing distinction between IncK1 and IncK2 plasmids. In this paper we examined the influence of these five polymorphisms in the RNAI structure for the compatibility of IncK1 and IncK2 plasmids. These results provide insights into the basis of incompatibility of IncK plasmids and support previous results for IncB/O plasmids.

## Methods

### Plasmids and vectors used

Plasmid pT.1.09 described in this study was recovered from *E. coli* from a poultry faeces sample and plasmid p754 was recovered from a dog faeces sample ( Table 1) ^22^. Vector pMW2 is a 4,4kb pBlueScript-derivative carrying the kanamycin resistance gene *aph(3')-III* ^24^.

**Table 1.** Plasmids used and constructed in this study.

Plasmid name	Resistance gene	Source	Reference
p754	*bla*_CTX-M-14_	dog	22
pT.1.09	bla _CMY-2_	poultry	22
pIncK1 mini	*aph(3')-III*	-	this study
pIncK2 mini	*aph(3')-III*	-	this study
pIncK1 mini RNAI_delA2	*aph(3')-III*	-	this study
pIncK1 mini RNAI_T10C	*aph(3')-III*	-	this study
pIncK1mini RNAI_G25T	*aph(3')-III*	-	this study
pIncK1 mini RNAI_G41C	*aph(3')-III*	-	this study
pIncK1 mini RNAI_G41C	*aph(3')-III*	-	this study
pIncK2 mini RNAI_insA2	*aph(3')-III*	-	this study
pIncK2 mini RNAI_G3C	*aph(3')-III*	-	this study
pIncK2 mini RNAI_C9T	*aph(3')-III*	-	this study
pIncK2 mini RNAI_T24G	*aph(3')-III*	-	this study
pIncK2 mini RNAI_C40G	*aph(3')-III*	-	this study

### Minireplicon construction

We examined the effect of point mutations in the RNAI structure on the compatibility of IncK plasmids. To determine the effect of the RNAI structure only and exclude involvement of any other plasmid structures, we designed minireplicons that contain the replication region of the IncK plasmid, and a kanamycin resistance cassette, which were ligated in a MW2 vector.

Minireplicons were constructed by cloning the replication region of the IncK1 or IncK2 plasmid into the vector pMW2. The replication region, corresponding to the one previously used for a minireplicon construction, contained repA, repB and RNAI ^25^. Amplifying the replication region was performed using the rep754 mini fw and rv primers for the IncK1 plasmid and repT1.09 mini fw and rv primers for IncK2 ( Table 2,). The PCR reaction was performed according to the protocol: 95°C 2 min, 95°C 30 sec, 57°C 30 sec, 72°C 2 min, 72°C 5min for 30 cycles. The PCR product was purified using the Gene Clean Turbo Kit (MoBio), digested with BamHI and KpnI for IncK1 and SacI and KpnI (Thermo Fisher Scientific) for IncK2. The digested replication region was ligated into vector MW2 after digestion with the respective enzymes, using T4 Ligase with standard manufacturers protocol (Thermo Fisher Scientific). The ligated product was electroporated into *E. coli* DH10B Electro MAX competent cells (Thermo Fisher Scientific) according to the manufacturer’s protocol. Transformants were grown on agar plates containing 25 µg/mL of kanamycin (Sigma). The presence of the minireplicon was confirmed by PCR using IncK replicon targeting primers (K1 fw and rv and K2 fw and rv) and Sanger sequencing. All created minireplicons are listed in Table 1. The size of the IncK1 minireplicon is 3789 bp and IncK2 3922 bp.

**Table 2.** Primers used in this study. “Mini” in the primer name means that the primer targets the minireplicon. Each primer name additionally depicts the mutation that is introduced in the RNAI structure, using this primer.

Primer name	Primer sequence	Reverse primer used	Reference
**Creating minireplicons**			
rep754 mini fw	CATGGTACCGGCCTGCAGTTCTGACAGAC		This study
rep754 mini rv	ATGTGATCATAGGCACGGTGCTGCGTTTG		This study
repT1.09 mini fw	CAGGGTACCACTGAGCCAGATACCAGTT		This study
repT1.09 mini rv	CAGGAGCTCTACGAGCGTGTACTGAGGAC		This study
**IncK1/IncK2 plasmids identification**			
K1 fw	ATCGTCAGGATCCGGGAAGTC		22
K1 rv	GAGCGATTGTGCCGTGTATT		22
K2 fw	ATGCTCGCGGTCCGGAAAGCC		22
K2 rv	GTGCCGTGCGTTAATGCACTGCAA		22
**Single nucleotide mutagenesis**			
Phu-754-R1	GGGATAAGTATATATGAAACCGTACCAGAG		This study
Phu-754-R2	TAGTAGGGGCGTTCACAGAATACGGGATAA		This study
Phu-T1.09-R1	GGGATAAGTATATATGAAACCGTGTCAGAG		This study
Phu-T1.09-R2	TAGTGGGGGCCTCACAGAATACGGGATAAG		This study
Phu-754-A2-Del-Fw	GTATTCTGTG ACGCCCCTACTATCTTTCACG	Phu-754-R1	This study
Phu-754-C4G-Fw	GTATTCTGTGAA GGCCCCTACTATCTTTCACG	Phu-754-R1	This study
Phu-754-T10C-Fw2	GTATTCTGTGAACGCCCC CACTATCTTTCACG	Phu-754-R1	This study
Phu-754-G25T-Fw	TCTTTCACGA TCCCGCCAAAGTTCGAGGAAAGAT	Phu-754-R2	This study
Phu-754-G41C-Fw	TCTTTCACGAGCCCGCCAAAGTTCGACGAAAGAT	Phu-754-R2	This study
Phu-T1.09-A2-Ins-Fw	GTATTCTGTG AAGGCCCCCACTATCTTTCACG	Phu-T1.09-R1	This study
Phu-T1.09-G3C-Fw	GTATTCTGTGA CGCCCCCACTATCTTTCACG	Phu-T1.09-R1	This study
Phu-T1.09-C9T-Fw	GTATTCTGTGAGGCCCC TACTATCTTTCACG	Phu-T1.09-R1	This study
Phu-T1.09-T24G-Fw	TCTTTCACGA GCCCGCCAAAGTTCGACGAAAGAT	Phu-T1.09-R2	This study
Phu-T1.09-C40G-Fw	TCTTTCACGATCCCGCCAAAGTTCGA GGAAAGAT	Phu-T1.09-R2	This study
**Resistance genes detection**			
Kan fw	ATGATGCTATGGCTGGAAGG		This study
Kan rv	CGCAGAAGGCAATGTCATAC		This study
CTX-M-14 fw	CTATTTTACCCAGCCGCAGC		26
CTX-M-14 rv	GTTATGGAGCCACGGTTGAT		26
CMY fw	ATGATGAAAAAATCGTTGCTGC		27
CMY rv	GCTTTTCAAGAATGCGCCAGG		27
aph(3')-III fw	GGCTAAAATGAGAATATCACCGG		28
aph(3')-III rv	CTTTAAAAAATCATACAGCTCGCG		28

Single nucleotide mutagenesis was performed with the Phusion Site-Directed Mutagenesis Kit (Thermo Scientific) according to the manufacturer’s protocol, using primers with the designated mutation ( Table 2). The presence of the minireplicon was confirmed with PCR using primers targeting the replication region ( Table 2). The presence of the mutation was confirmed by Sanger sequencing.

The RNAI structure of the wild type (wt) and mutated RNAI genes was predicted using RNAfold ( http://rna.tbi.univie.ac.at/cgi-bin/RNAWebSuite/RNAfold.cgi).

### Stability

The stability of minireplicons was determined in triplicate by independent overnight culturing in LB broth without selection. Serial dilutions of the culture were plated on LB agar plates without selection and incubated overnight at 37°C. 40 colonies were picked and PCR was performed to amplify the resistance gene present on the minireplicon. Stability was determined as a percentage of colonies containing the minireplicon. All results were statistically analysed using the Mann–Whitney U test.

### Plasmid copy number

To determine the plasmid copy number, three independent DNA extractions using DNeasy UltraClean Microbial Kit (Qiagen) were performed from overnight culturing in LB broth with selection for each strain, and qPCRs using iQ SYBR Green Supermix (BioRad), targeting the *aph(3’’)-III* replicon and *uidA* gene, were carried out in triplicate for each extraction. Plasmid copy number per chromosome was calculated using the formula described by San Millan *et al.* ^29^ cn = [(1 + Ec) ^Ctc^/(1 + Ep) ^Ctp^] x (Sc/Sp), where cn is the plasmid copy number per chromosome, Sc and Sp are the sizes of the chromosomal and plasmid amplicons (in bp), respectively, Ec and Ep are the efficiencies of the chromosomal and plasmid qPCRs (relative to 1), respectively, and Ctc and Ctp are the threshold cycles of the chromosomal and plasmid reactions, respectively. Plasmid copy number was determined using aph(3’’)-III fw and aph(3’’)-III rv primers for IncK1 minireplicons and uidA fw and uidA rv for the chromosomal target ( Table 2). Obtained data were analysed using the Mann–Whitney U test.

### Incompatibility testing

IncK minireplicons are non-conjugative, therefore electroporation was chosen as a method to deliver the minireplicons into the bacterial cell. IncK1 or IncK2 wt plasmids were electroporated into *E. coli* DH10B according to the manufacturer’s protocol. *E. coli* cells carrying either the IncK1 or IncK2 wt plasmid were made electrocompetent from 250 mL liquid culture of OD _600 _0.5 in LB media. Cultures were spun down for 10 min at 3560× *g* at 4°C. Pellets were washed twice with 250 and 125 mL ice-cold water, washed with 10 mL ice-cold 10% glycerol and finally resuspended in 0.5 mL ice-cold 10% glycerol and frozen at −80°C. For electroporation, minireplicons were isolated using the Wizard Plus SV Miniprep kit (Promega) and transformed as described above. Transformants were subsequently selected on LB plates supplemented with 25 µg/mL kanamycin (Sigma-Aldrich) to select for the minireplicon and 2 µg/mL cefotaxime (Sigma-Aldrich) to select for the IncK wt plasmid.

To test the incompatibility of the IncK plasmid and the minireplicon combinations, the heteroplasmid population was grown overnight and plated on non-selective LB agar plates. For a detailed description see the “stability” paragraph.

## Results

### RNAI structure comparison

**Figure 1.**
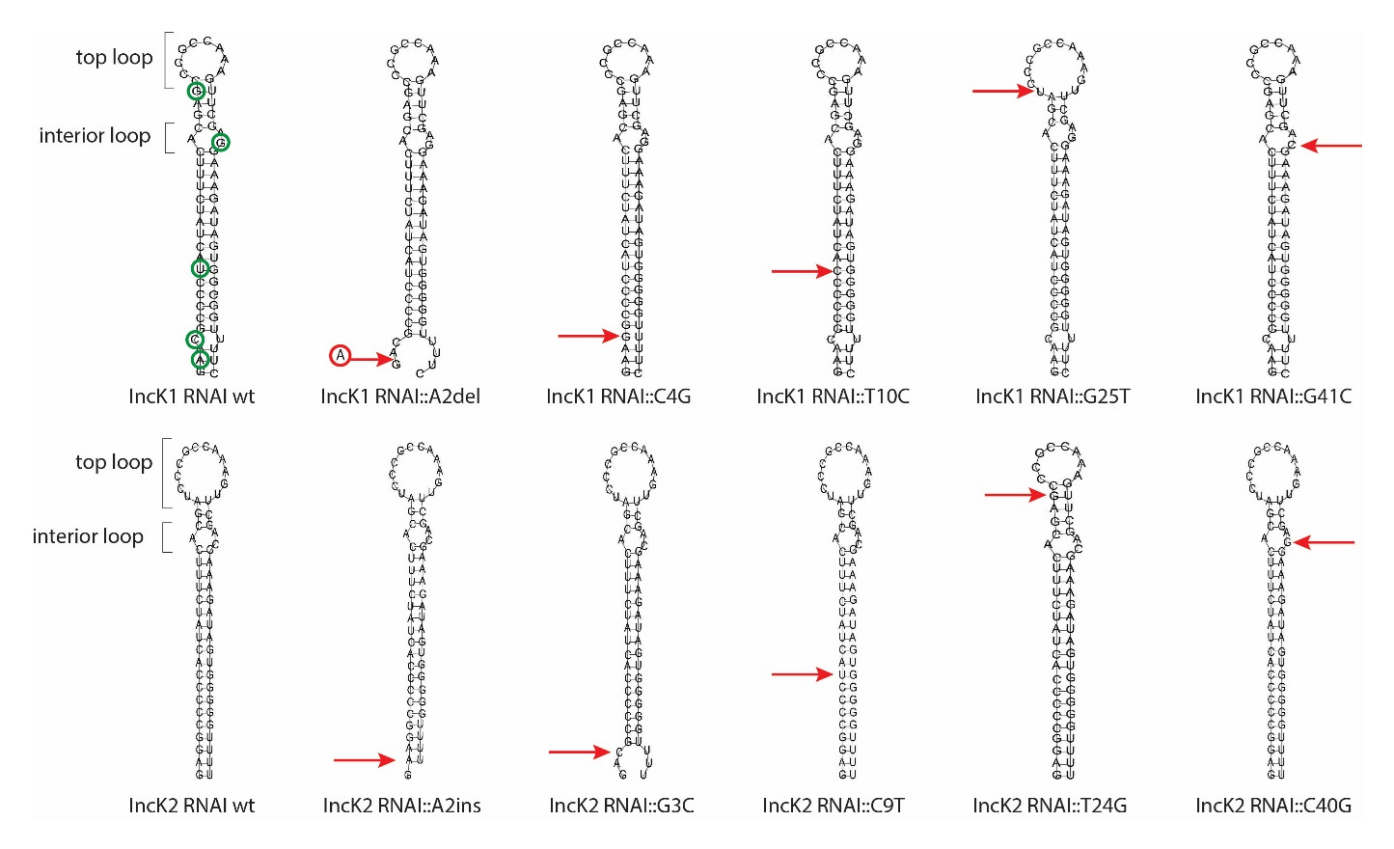
RNA structure prediction of all mutated RNAI variants. Red arrows point out the different mutation sites of the various minireplicons that were tested. Green circles indicate all SNPs between IncK1 and IncK2 RNAI.

The RNAI sequence of IncK1 and IncK2 sequences differ by four SNPs and one indel. The RNAI structures of the wt plasmids were predicted as well as the structures of variants where one polymorphism of IncK2 is introduced in IncK1 and vice versa ( Figure 1). The RNAI structures of wt IncK1 and IncK2, which consist of 61 and 60 nucleotides respectively, mainly differ in the top loop region. The top loop of the IncK2 RNAI structure is substantially bigger (12 nucleotides) compared to the one from IncK1 (8 nucleotides) based on *in silico* RNA structure predictions. The predicted size of the loop is controlled by a single polymorphism at the base of the loop while all other polymorphisms affect the stem or interior loop of the molecule.

All of the nucleotide positions that are different between IncK1 and IncK2 plasmids were subjected to site-directed mutagenesis. Each mutated nucleotide was substituted with the corresponding nucleotide from the opposite plasmid group. All of the mutations have an effect on the predicted structure, either creating or dissolving the second interior loop at the base of the stem or creating an overhang of several ‘free’ bases. However, mutation G25T/T24G is the only mutation that affects the top loop and therefore has the biggest impact on the conformation of the predicted structure ( Figure 1).

### Minireplicon stability

**Figure 2.**
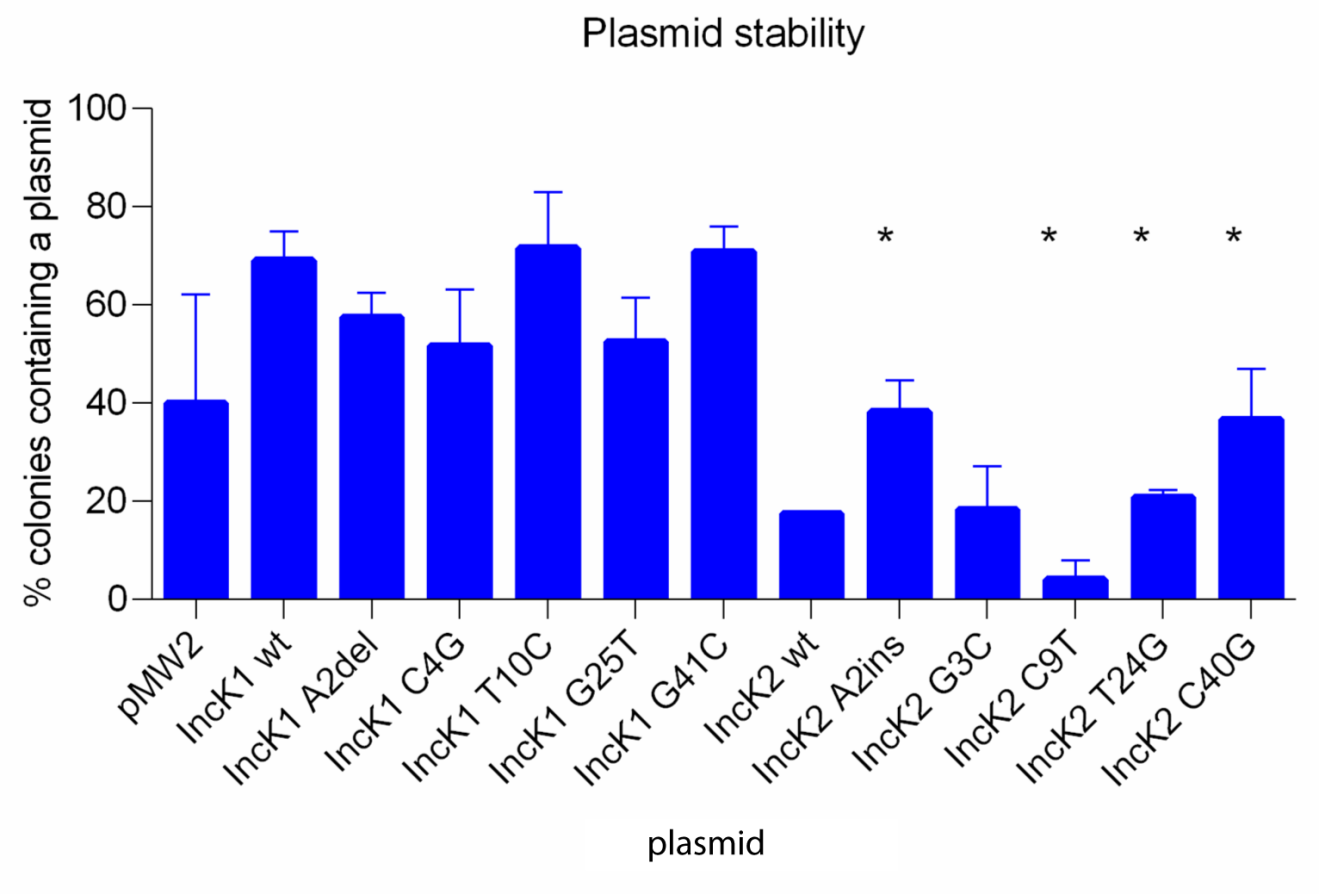
Stability of wt and mutant minireplicons after 24 hours of non-selective growth. Asterisks indicate the mutant minireplicons for which stability was significantly different compared to the minireplicons containing the wt RNAI (p≤0.05).

The stability of the minireplicons as well as the vector pMW2 was examined in triplicate after 24 hours of culture without antibiotic selection and was defined as the percentage of colonies that contained the minireplicon at the end of experiment. Stability of the vector pMW2 was 40%, which is lower compared to IncK1 minireplicons ( Figure 2). However, the difference between stability of pMW2 and any minireplicon was not statistically significant, probably due to high standard deviation. The IncK1 wt minireplicon had a higher stability compared to the IncK2 wt minireplicon (p=0.034). All IncK1 mutated minireplicons have a higher stability than the corresponding IncK2 mutated minireplicons (p≤0.05). For IncK1 plasmids there were no statistically significant differences between the wt minireplicon and mutated variants ( Figure 2). For IncK2 minireplicons, mutations A2ins, T24T and C40G resulted in a statistically significant increase in stability of the minireplicon compared to wt (p=0.037 for A2ins and C40G and p=0.034 for T24T). On the other hand, mutation C9T caused a statistically significant decrease in stability of the minireplicon compared to the IncK2 wt (p=0.037). This low stability and high variability of the IncK2-derived minireplicons may affect the results and IncK2-derived minireplicons were therefore excluded from plasmid copy number and incompatibility testing.

### Plasmid copy number

**Figure 3.**
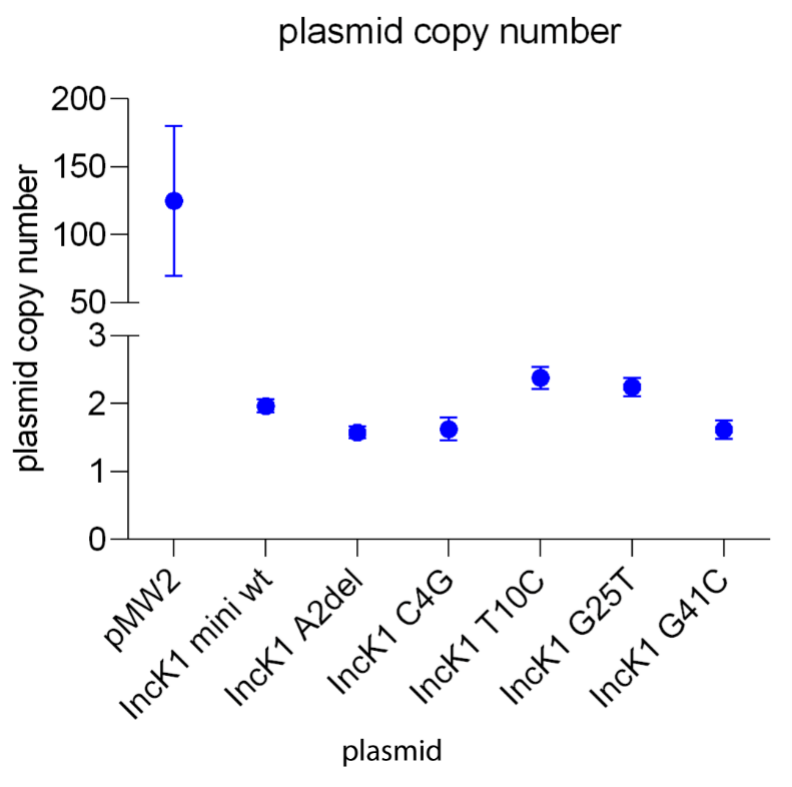
Plasmid copy number.

Plasmid copy number was determined using qPCR targeting *aph(3’’)-III* as a plasmid target gene and *uidA* as a genomic target gene. Vector pBlueScript, which is a backbone for the vector pMW2, is a high-copy number plasmid which was confirmed in our experiment. pMW2 has an average copy number of 125. The IncK1-based minireplicons have an average copy number of 2. This result corresponds to the previous reports showing that copy number of IncK1 wt plasmids is 1–2 copies per cell ^30^. For all of the minireplicons with mutated RNAI there is no significant difference in copy number compared to the wt minireplicon ( Figure 3).

### Minireplicon incompatibility

Incompatibility of IncK1 wt-derived mutated minireplicons was checked in triplicate against the parental and non-parental IncK wt plasmids. We performed overnight incompatibility tests to assess if the tested minireplicons and wt plasmids could be stably present together in one cell without antibiotic selection.

**Figure 4.**
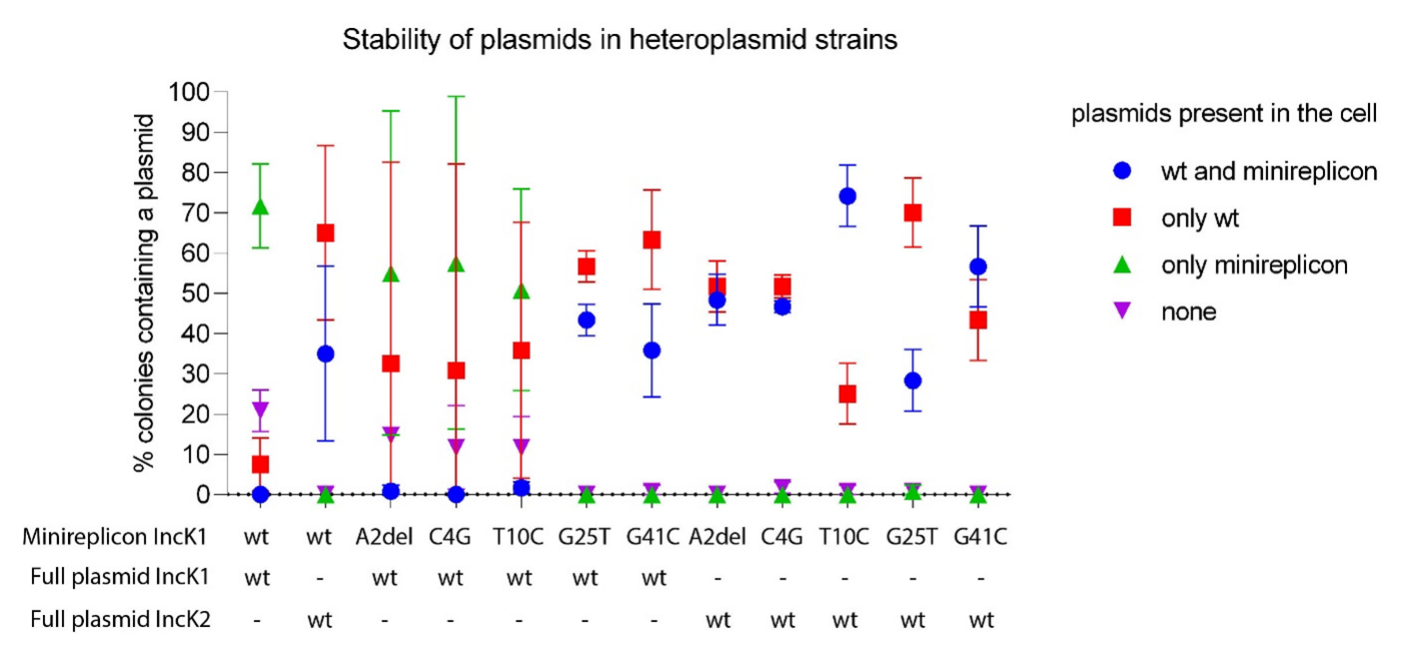
Compatibility of wt and mutated minireplicons tested in triplicate against parental and non-parental IncK plasmids. Raw data can be found in Supplementary Table 1.

All heteroplasmid strains were created by electroporating a mutated minireplicon with wt IncK1 or IncK2 plasmid into one cell. The compatibility of plasmids from all obtained heteroplasmid cells are shown in Figure 4. We determined the percentage of cells carrying either both a minireplicon and IncK wt plasmid, only one of these or none. All minireplicons were compatible with their non-parental IncK wt plasmid ( Figure 4, Extended data ^31^, Table S1). Mutations G25T and G41C have a critical effect on compatibility of the IncK1 minireplicon with the IncK1 wt parental plasmid. Mutations A2del and T10C also cause some degree of compatibility with the IncK1 parental plasmid.

**Figure 5.**
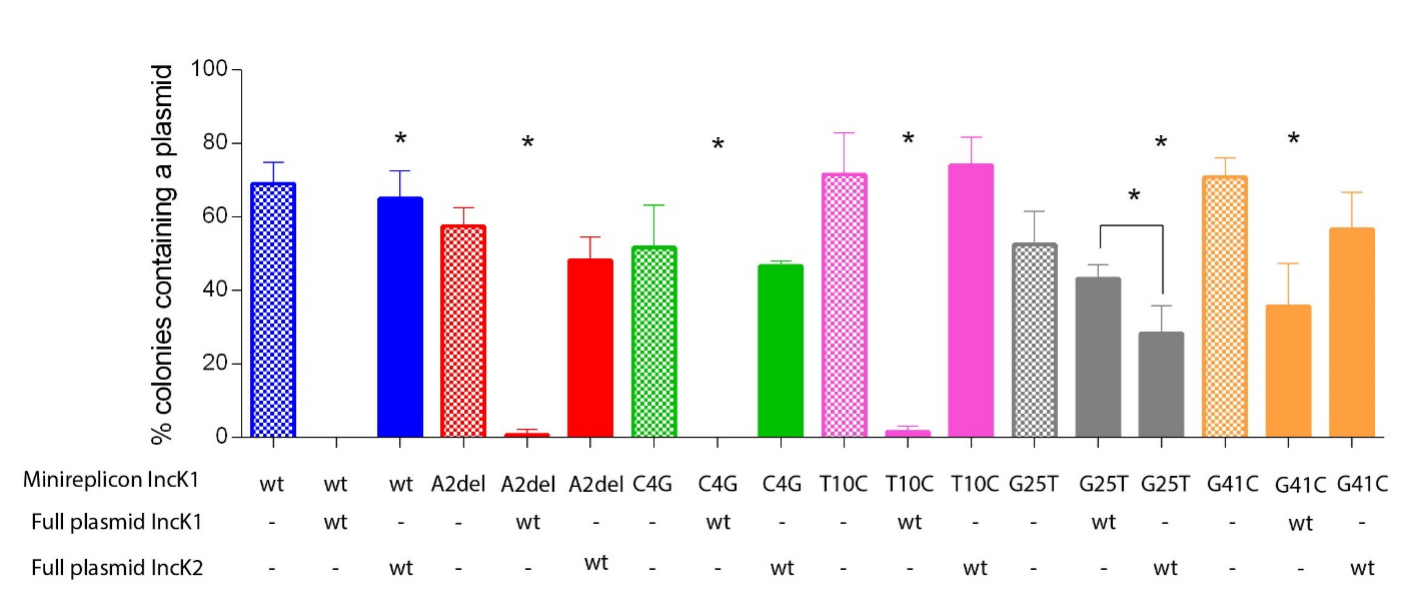
Comparison of single minireplicon stability with its stability in the heteroplasmid strain (co-existing with parental or non-parental plasmids). Sample names under the graph show minireplicons used before the “/” and IncK wt plasmids used after “/”. Dotted bars represent stability of the minireplicon alone, full bars represent stability of minireplicons with the IncK1 wt plasmid. * Means that the minireplicon stability in the heteroplasmid sample is statistically significantly different from the stability of a single minireplicon.

Based on our incompatibility experiments, we compared the stability of single minireplicons to the plasmids from heteroplasmid strains containing both the minireplicon and their (non-) parental IncK wt plasmids ( Figure 5). All minireplicons that co-existed with their parental plasmid had a statistically significant lower stability in comparison with plasmids from cells containing only the minireplicon ( Figure 4). The only exception is mutation G25T on the IncK1 minireplicon, where there was no statistical difference in stability between the heteroplasmid or single plasmid strains. This is also the only mutation that restored full compatibility of the minireplicon and its parental plasmid. Mutation G25T on the IncK1 minireplicon resulted in a decreased compatibility with its non-parental plasmid. 

## Discussion

In this study we examined the molecular basis of incompatibility of IncK plasmids by determining the effect of point mutations in the RNAI structure on incompatibility. We created minireplicons carrying the replication region of either the IncK1 or IncK2 plasmid, in which we subsequently introduced point mutations in the RNAI structure.

The minireplicons constructed in this study consisted of the replication region of IncK1 or IncK2 plasmids and an *aph(3’’)-III* gene. The copy number of the plasmid is regulated by the replication region. IncK wt plasmids have been shown to have a copy number of 1–2 per cell ^30^. Lack of the partitioning system on the low-copy minireplicons leads to random segregation during cell division and therefore rapid plasmid loss. Additionally, the IncK wt plasmid has a toxin-antitoxin system stabilizing it in a cell, which is missing on the minireplicons. This could explain the instability of the minireplicons created in this study. High instability of minireplicons was previously reported for IncL/M plasmids ^32^. Additionally, the fact that the minireplicons constructed in this study lack the partitioning system may also contribute to why the observed incompatibility was weaker than expected. The obtained results indicate that replication-based incompatibility may not be a straight-forward system based on plasmids being present or not in one cell. It may be similar to partitioning-based incompatibility of IncF plasmids, where both strong and weak incompatibility was reported ^33^.

The results obtained in this study showed that mutations in the top loop and interior loop in the upper stem of the RNAI structure have a critical effect on the compatibility of IncK plasmids. Similar findings were previously demonstrated for IncB/O plasmids ^12^. Three nucleotides in the top loop of RNAI of IncB/O plasmid structure, G37, C38, C39, have the most significant effect on the compatibility of IncB/O plasmids, because they are responsible for the formation of the initial kissing complex between RNAI and SLI ^12^. Mutations in the bottom part of the top loop of IncB/O plasmid have less significant effect on compatibility, which would explain the results that heteroplasmid strains containing IncK wt plasmid and IncK minireplicon with the top loop mutation, G25T for IncK1 were compatible. The interior loop in the upper stem of RNAI is involved in inter-strand pairing between RNAI and SLI ^21^ Preserving the structure of the interior loop is crucial for the interaction with SLI. Mutation G41C, that was introduced into RNAI in this study, does not disrupt the interior loop and therefore has a limited effect on the incompatibility of IncK plasmids. Additionally, different mutations cause a different degree of incompatibility for IncK1 and IncK2 plasmids. For IncK1 minireplicons, mutations G25T and G41C have a critical effect on their compatibility with the IncK1 wt parental plasmid. Mutations A2del and T10C only have a small effect on the compatibility. These changes in compatibility of IncK1 minireplicons could be observed regardless its low stability. However, mutation T10C causes the highest increase in the copy number from all of the created mutations. These results implicate that mutation T10C does not affect compatibility of IncK1 and IncK2 plasmids, but has an effect on replication and copy number control of IncK1 plasmid. It is possible that exchanging thymine to cytosine results in stronger bonding with guanine on the opposite strands which interferes with the formation of the extended kissing complex. These findings are in line with previous research, which showed that different mutations in the RNAI structure can cause a different degree of *rep* inhibition ^12^. Incompatibility of IncK plasmids can be caused by the cumulative effect of the mutations in the RNAI structure. However, low stability of the minireplicons may be the sole reason why not all initially heteroplasmid strains contain both plasmids. More research is required to be able to fully understand the role of mutations in RNAI and the interplay of partitioning system in the compatibility of these plasmids.

In recent years, compatibility of many plasmids was re-examined ^22, 23, 34, 35^. The most used and well established method to determine compatibility of two plasmids is conjugation of both into one cell, followed by selective plating. However, the present study has indicated the added value of examining plasmid incompatibility over time in comparison with the classical method where incompatibility is measured as an ability to form a heteroplasmid strain. This greatly affected the interpretation of results. Incompatible plasmids could be found in one cell with selective pressure and were unstable together after removal of the selective pressure.

Based on previous sequence alignments, RNAI-structure predictions and minireplicon incompatibility experiments, this manuscript has confirmed the predicted effect of certain bases in the RNAI structure of IncK1 and IncK2 plasmid incompatibility. It shows that a single mutation in the RNAI structure of the IncK1 plasmid can change its compatibility. The RNAI structures used in this study were predicted *in silico*. However, using crystallographic RNAI structures could provide a better resolution and insights about the exact mechanism underlying the compatibility changes caused by the introduced mutations. Further research in this field, including more plasmid types, can possibly lead to a design of a sequence-based tool predicting plasmid compatibility. Such a tool would eliminate the necessity to perform laborious compatibility experiments and allow faster and easier plasmid compatibility predictions. Knowledge about plasmids compatibility would allow to improve existing plasmid classification, which would further help understanding epidemiology of these plasmids.

## Data Availability

Biostudies: Underlying and extended data for “Understanding the genetic basis of the incompatibility of IncK1 and IncK2 plasmids.” https://www.ebi.ac.uk/biostudies/studies/S-BSST977 This project contains the following underlying data: Data file 1. Raw data underlying figures 2, 3, 4, and 5 and extended data. ENA: Sequencing data of the strains used in this study https://www.ebi.ac.uk/ena/browser/view/ERR1551799 https://www.ebi.ac.uk/ena/browser/view/ERR1607717
