# Supplementary material for: Understanding the genetic basis of the incompatibility of IncK1 and IncK2 plasmids
Source: Open Res Eur. 2023 Oct 30;3:53. Originally published 2023 Apr 11. [Version 2] doi: 10.12688/openreseurope.15121.2 (PMC10724649; doi:10.12688/openreseurope.15121.2)
Supplement: Peer review history

# Reviewer report: Reviewer response for version 2

**DOI:** 10.21956/openreseurope.18124.r35951

**Published:** 2023-12-15

**Authors:** Lu Yang

**Affiliations:** 1 Shanghai University of Sport, Shanghai, China; 2 China Agricultural University, Beijing, Beijing, China

I do not have further comments to make.

Is the study design appropriate and does the work have academic merit?

Yes

Is the work clearly and accurately presented and does it cite the current literature?

Yes

If applicable, is the statistical analysis and its interpretation appropriate?

Yes

Are all the source data underlying the results available to ensure full reproducibility?

Yes

Are the conclusions drawn adequately supported by the results?

Yes

Are sufficient details of methods and analysis provided to allow replication by others?

Yes

Reviewer Expertise:

Microbiology, Antimicrobial resistance, Genomics

I confirm that I have read this submission and believe that I have an appropriate level of expertise to confirm that it is of an acceptable scientific standard.

# Reviewer report: Reviewer response for version 2

**DOI:** 10.21956/openreseurope.18124.r35952

**Published:** 2023-11-16

**Authors:** Manuel Ares-Arroyo

**Affiliations:** 1 Department of Genomes and Genetics, Institut Pasteur, Paris, Île-de-France, France

I do not have further comments to make.

Is the study design appropriate and does the work have academic merit?

Yes

Is the work clearly and accurately presented and does it cite the current literature?

Yes

If applicable, is the statistical analysis and its interpretation appropriate?

Yes

Are all the source data underlying the results available to ensure full reproducibility?

Yes

Are the conclusions drawn adequately supported by the results?

Yes

Are sufficient details of methods and analysis provided to allow replication by others?

Yes

Reviewer Expertise:

Plasmid biologist. Mobile Genetic Elements. Bacterial evolution.

I confirm that I have read this submission and believe that I have an appropriate level of expertise to confirm that it is of an acceptable scientific standard.

# Reviewer report: Reviewer response for version 1

**DOI:** 10.21956/openreseurope.16358.r33419

**Published:** 2023-08-02

**Authors:** Lu Yang

**Affiliations:** 1 Shanghai University of Sport, Shanghai, China; 2 China Agricultural University, Beijing, Beijing, China

The manuscript titled "Understanding the genetic basis of the incompatibility of IncK1 and IncK2 plasmids" by Rozwandowicz et al. aims to investigate the genetic basis of incompatibility between IncK1 and IncK2 plasmids in bacteria. Overall, the study is well-conducted and holds valuable insights into the dynamics of plasmid interactions within bacterial cells. However, I have some concerns and suggestions that should be addressed before the manuscript can be considered for indexing. Please find my specific comments below:

  

1. The stability of the vector pMW2 is reported to be 40% and widely dispersed, but its potential impact on the study's results is not clearly addressed. I recommend the author to provide a more detailed description of the experimental design and explain why to choose this vector and competent cells in this study. I also suggest the author include a brief discussion in the manuscript's conclusion about how the low stability of the vector might affect the experimental outcomes. Addressing this issue would enhance the scientific rigor and credibility of the study, ensuring a more cautious interpretation of the results.
2. In the manuscript, there appears to be no direct comparison of the incompatibility of IncK2 mutated minireplicons with the IncK1 wild-type plasmids. This omission limits the comprehensive understanding of the plasmid's behavior and compatibility between these two types. It would greatly benefit the readers and the significance of the study if you could address this issue.
3. To enhance its significance, I recommend explicitly discussing the relationship between this type of plasmid and antibiotic resistance. This will strengthen the manuscript's impact on the field.

Is the study design appropriate and does the work have academic merit?

Yes

Is the work clearly and accurately presented and does it cite the current literature?

Yes

If applicable, is the statistical analysis and its interpretation appropriate?

Yes

Are all the source data underlying the results available to ensure full reproducibility?

Yes

Are the conclusions drawn adequately supported by the results?

Yes

Are sufficient details of methods and analysis provided to allow replication by others?

Yes

Reviewer Expertise:

Microbiology, Antimicrobial resistance, Genomics

I confirm that I have read this submission and believe that I have an appropriate level of expertise to confirm that it is of an acceptable scientific standard, however I have significant reservations, as outlined above.

# Response

**Published:** 2023-10-23

**Authors:** Marta Rozwandowicz

**Affiliations:** RIVM, The Netherlands

**Lu Yang** The manuscript titled "Understanding the genetic basis of the incompatibility of IncK1 and IncK2 plasmids" by Rozwandowicz et al. aims to investigate the genetic basis of incompatibility between IncK1 and IncK2 plasmids in bacteria. Overall, the study is well-conducted and holds valuable insights into the dynamics of plasmid interactions within bacterial cells. However, I have some concerns and suggestions that should be addressed before the manuscript can be considered for indexing. Please find my specific comments below:  

1. The stability of the vector pMW2 is reported to be 40% and widely dispersed, but its potential impact on the study's results is not clearly addressed. I recommend the author to provide a more detailed description of the experimental design and explain why to choose this vector and competent cells in this study. I also suggest the author include a brief discussion in the manuscript's conclusion about how the low stability of the vector might affect the experimental outcomes. Addressing this issue would enhance the scientific rigor and credibility of the study, ensuring a more cautious interpretation of the results.

  - The reason for choosing pMW2 vector was the presence of kanamycin resistance gene and *E. coli* DH10B Electro MAX were the authors’ choice because of their high transformation efficiency. Both arguments are now included in the methods under the “Minireplicon construction” paragraph. The discussion about the impact of pMW2 vector’s low stability is now included in the discussion part of the manuscript.  

1. In the manuscript, there appears to be no direct comparison of the incompatibility of IncK2 mutated minireplicons with the IncK1 wild-type plasmids. This omission limits the comprehensive understanding of the plasmid's behavior and compatibility between these two types. It would greatly benefit the readers and the significance of the study if you could address this issue.

  - The authors believe that due to the very low stability of IncK2 minireplicons such an experiment would not yield reliable results. An appropriate comment is included at the end of the “Minireplicon stability” paragraph of the results section.  

1. To enhance its significance, I recommend explicitly discussing the relationship between this type of plasmid and antibiotic resistance. This will strengthen the manuscript's impact on the field.

  - Resistance genes most commonly associated with IncK1 and IncK2 plasmids, as well as the sources where these plasmids are found are now discussed in the introduction part of the manuscript. Additionally, a strong association of IncK2 plasmids to poultry sources is mentioned. **Is the work clearly and accurately presented and does it cite the current literature?** Yes **Is the study design appropriate and does the work have academic merit?** Yes **Are sufficient details of methods and analysis provided to allow replication by others?** Yes **If applicable, is the statistical analysis and its interpretation appropriate?** Yes **Are all the source data underlying the results available to ensure full reproducibility?** Yes **Are the conclusions drawn adequately supported by the results?** Yes ***Competing Interests: *** No competing interests were disclosed. ***Reviewer Expertise: *** Microbiology, Antimicrobial resistance, Genomics **I confirm that I have read this submission and believe that I have an appropriate level of** **expertise to confirm that it is of an acceptable scientific standard, however I have** **significant reservations, as outlined above.**

# Reviewer report: Reviewer response for version 1

**DOI:** 10.21956/openreseurope.16358.r33413

**Published:** 2023-07-20

**Authors:** Manuel Ares-Arroyo

**Affiliations:** 1 Department of Genomes and Genetics, Institut Pasteur, Paris, Île-de-France, France

The aim of the work is to characterize the molecular basis of incompatibility within and between two related subgroups of plasmids from the I-complex, the IncK1 and IncK2. Combining an *in silico* analysis and a molecular biology approach, the authors show how just one SNP in the RNAI of these plasmids turn them into compatible replicons. Despite other features of the plasmids are not tested (e.g., the partitioning system), this represents an interesting piece of work for plasmid biologists in particular, which shows how replication-mediated incompatibility works in two related lineages of plasmids.

 In general terms, there are not big concerns regarding the bibliography, reproducibility, statistical analysis, etc. Notwithstanding, there are few concerns I would like to comment regarding the present manuscript that make me consider the work Approved With Reservations:

**General concerns:**

**Stability of pT.109 and p754**. It seems that the authors did not test the stability of the wt IncK1 (pT.109) and IncK2 (p754), or data is not shown. I find a bit disturbing the result in Figure 4, in which the incompatibility between the minireplicon IncK1 and the full plasmid IncK1 results in the loss of the full wt plasmid: 70% of the colonies carry the minireplicon, whereas less than 10% carry the full wt plasmid. According to the authors, these plasmids encode for a partitioning system in addition to a toxin/antitoxin system. Given the expected stability of this plasmid, isn’t it unexpected that the minireplicon is the one that is mostly maintained in the population after an overnight? How stable are these wt plasmids in E. coli DH10B? Any comments about this result?

**Incompatibility between pT.109 and p754**. The authors tested the incompatibility between the minireplicons with the full wt plasmids. However, at no point the authors tested the incompatibility between the two full wt plasmids. Did the authors calculate it? Does it show similar results to the minireplicon IncK1 with the full plasmid IncK2? Likewise, it could help to understand to which extent other factors could be implicated in the plasmid incompatibility, commented at some points within the text.

**Temperature (37ºC and 42ºC)**: IncK1 and IncK2 usually inhabit niches with different temperatures, being IncK2 plasmids specially adapted to temperatures of 42ºC (poultry temperature). It was shown by authors of this work that both IncK1 and Inck2 lineages show different plasmid copy number, conjugation rates and could have different fitness cost depending on this temperature (Rozwandowicz et al., 2019, Frontiers Microbiology) ^1^. Since all the experiments were done at 37ºC, it is not unexpected to find that the stability of IncK2-derivatives is lower than that of IncK1-derivatives. Did the authors test if the IncK2 minireplicons at 42ºC have a stability comparable to the IncK1 at 37ºC, so that they are not discarded from the work? Did the authors tested different temperatures?

**Specific concerns:**

**Introduction**.

**1 ^st^ Paragraph**. It states that “there are 40 plasmid types described, mostly based on their incompatibility”. There are 40 types according to the incompatibility, but additional classification methods are being used (MOB, MPF, PTUs,…) and they not included in these 40 aforementioned types. I would change ‘mostly based on’ by ‘according to’.

**2 ^nd^ Paragraph. **It could be convenient a reference to the definition of incompatibility.

**3 ^rd^ Paragraph**. The authors mention that plasmid replication regulated by antisense RNA occurs in IncF, IncL, IncM and I-complex plasmids. However, within the references cited (5-11), there is also the plasmid R1162 (reference 9, which is an incQ) and the gram positive plasmid pT181 (reference 5, typed as rep7a). Additionally, ColE1 replication could be added to the list.

**4 ^th^ Paragraph**. A schematic figure representing the replication mechanism of I-complex plasmids (with the role of RNAI) might be useful for readers not used to this plasmid complex. The second half of the **5 ^th^ paragraph** could also benefit of the figure. Additionally, it could include the general genetic map of the minireplicons used (present in the extended data).

**Methods**.

**Plasmids and vectors used.** Either in the text and/or in the table could be indicated which wt plasmid is IncK1 or Inck2 (pT.109 or p754).

**Incompatibility testing. **The minireplicons were electroporated because they are non-conjugative, the authors wrote. But, the full wt plasmids were electroporated as well (next sentence). Are they also non-conjugative? What is their size? No information on the manuscript.

**Results**.

**RNAI structure comparison**. The last sentence of the 1 ^st^ paragraph (“The predicted size… …interior loop of the molecule”) could fit better in the second paragraph, with the result of G25T/T24G, or at least after mentioning that some substitutions were tested.

**Figure 1.** The RNAfold server has the option to color the nucleotides according to the base-pair probability. Have the authors considered the option to include the probabilities in the secondary structure inference? Do the wt RNAI’s secondary structures show high probabilities? And the mutated ones?

**Minireplicon stability.** Does the minireplicons’ stability correlate with the one of the wt original plasmids? Is pT.109 more stable than p754 in these conditions?

**Minireplicon stability.** Some SNPs in the RNAI of IncK2 increase/decrease the stability, but the secondary structure (in Figure 1) is really conserved. Do the base-pair probabilities of these structures change?

**Minireplicon incompatibility**. ‘…against the parental and non-parental IncK wt plasmids’ means against pT.109 and p754?

**Figure 4. **For the representation of this Figure, might the authors benefit from a stacked histogram.

**Discussion.**

**1 ^st^ Paragraph. **In addition to the RNAI differences, how similar are the rep genes? Could these rep genes also provide some incompatibility? Additionally, how conserved are these RNAI in nature? All IncK1 and IncK2 plasmids have the exact same RNAIs or are there differences dissimilarities in between wt plasmids?

**2 ^nd^ Paragraph.** If full wt plasmids should be more stable (toxin/antitoxin, partitioning), one would expect them to be more stable when assessing the compatibility, not being more loss than the minireplicon (as it is in Figure 4). Any explanation? Are these full wt plasmids more stable than the minireplicons? No data on this aspect in the text.

**2 ^nd^ Paragraph.** “The observed incompatibility is weaker than expected”. What were the expectation of the authors?

**2 ^nd^ Paragraph.** The authors state that “the results indicate that replication-based incompatibility may not be a straight-forward system based on plasmids being present in one cell.” But indeed, this is exactly what is occurring in the experiment. Among the bacteria carrying both the full IncK1 and the minireplicon IncK1, 0% of the colonies tested carry both plasmids after just one overnight. Despite other plasmid characteristics may be involved, it does suggest that incompatibility is infeluncing the compatibility via replication-based mechanisms.

**3 ^rd^ Paragraph**. Full stop (“.”) missing between reference 21 and the next sentence (“Preserving the structure…”).

**3 ^rd^ Paragraph**. “T10C causes the highest increase in plasmid copy number”. In results, it was shown that there are no statistical differences in copy number between the IncK1 minireplicons.

**4 ^th^ Paragraph**. Do the authors have references for ‘the most used method to determine compatibility of two plasmids is conjugation?’

Is the study design appropriate and does the work have academic merit?

Yes

Is the work clearly and accurately presented and does it cite the current literature?

Yes

If applicable, is the statistical analysis and its interpretation appropriate?

Yes

Are all the source data underlying the results available to ensure full reproducibility?

Yes

Are the conclusions drawn adequately supported by the results?

Yes

Are sufficient details of methods and analysis provided to allow replication by others?

Yes

Reviewer Expertise:

Plasmid biologist. Mobile Genetic Elements. Bacterial evolution.

I confirm that I have read this submission and believe that I have an appropriate level of expertise to confirm that it is of an acceptable scientific standard, however I have significant reservations, as outlined above.

## References

[1] : Successful Host Adaptation of IncK2 Plasmids. *Front Microbiol* .2019;10: 10.3389/fmicb.2019.02384 2384 10.3389/fmicb.2019.02384 31681238 PMC6803427

# Response

**Published:** 2023-10-23

**Authors:** Marta Rozwandowicz

**Affiliations:** RIVM, The Netherlands

***Manuel Ares-Arroyo*** *Institut Pasteur Departement de Virologie, Paris, Île-de-France, France* *The aim of the work is to characterize the molecular basis of incompatibility within and between* *two related subgroups of plasmids from the I-complex, the IncK1 and IncK2. Combining an in silico* *analysis and a molecular biology approach, the authors show how just one SNP in the RNAI of* *these plasmids turn them into compatible replicons. Despite other features of the plasmids are* *not tested (e.g., the partitioning system), this represents an interesting piece of work for plasmid* *biologists in particular, which shows how replication-mediated incompatibility works in two* *related lineages of plasmids.* *In general terms, there are not big concerns regarding the bibliography, reproducibility, statistical* *analysis, etc. Notwithstanding, there are few concerns I would like to comment regarding the* *present manuscript that make me consider the work Approved With Reservations:* ***General concerns:*** ***Stability of pT.109 and p754*** *. It seems that the authors did not test the stability of the wt IncK1* *(pT.109) and IncK2 (p754), or data is not shown. I find a bit disturbing the result in Figure 4, in* *which the incompatibility between the minireplicon IncK1 and the full plasmid IncK1 results in the* *loss of the full wt plasmid: 70% of the colonies carry the minireplicon, whereas less than 10% carry* *the full wt plasmid. According to the authors, these plasmids encode for a partitioning system in* *addition to a toxin/antitoxin system. Given the expected stability of this plasmid, isn’t it* *unexpected that the minireplicon is the one that is mostly maintained in the population after an* *overnight? How stable are these wt plasmids in E. coli DH10B? Any comments about this result?*

 Stability of IncK1 and IncK2 plasmids was not performed in the present study, however, It was checked in previous study describing compatibility of these plasmids (Rozwandowicz et al. 2017). In Rozwandowicz et al. 2017 stability of IncK1 or IncK2 plasmid was determined in the cell carrying both IncK1 and IncK2 plasmids. After 72 h, 98% of the IncK1 plasmids were still present using selective agar and 100% using nonselective agar. The IncK2 plasmid showed 100% stability on both selective and nonselective agar. An appropriate sentence was added to the “Minireplicon stability” part of the results.Obtained results are actually not that surprising considering that plasmid incompatibility was initially defined as a displacement of a resident plasmid (in this case wild type IncK plasmid). Moreover, in a direct competition, the authors predict that the minireplicons have a lower fitness cost compared to the wt plasmid.

***Incompatibility between pT.109 and p754*** *. The authors tested the incompatibility between the* *minireplicons with the full wt plasmids. However, at no point the authors tested the* *incompatibility between the two full wt plasmids. Did the authors calculate it? Does it show similar* *results to the minireplicon IncK1 with the full plasmid IncK2? Likewise, it could help to understand* *to which extent other factors could be implicated in the plasmid incompatibility, commented at* *some points within the text.*

 The incompatibility of two wt plasmids is not presented in this manuscript, but it was demonstrated in previous work from the authors (Rozwandowicz et al. 2017). Although the exact pair used in this study was not tested previously, authors believe that the results can be extrapolated. The results presented in the previous study correlate nicely with the current study. In the pair of IncK2 wt and IncK1 minireplicon, the InK2 plasmid is nearly 100% stable. An appropriate sentence was added to the “Minireplicon incompatibility” part of the results.

***Temperature (37ºC and 42ºC)*** *: IncK1 and IncK2 usually inhabit niches with different* *temperatures, being IncK2 plasmids specially adapted to temperatures of 42ºC (poultry* *temperature). It was shown by authors of this work that both IncK1 and Inck2 lineages show* *different plasmid copy number, conjugation rates and could have different fitness cost depending* *on this temperature (Rozwandowicz et al., 2019, Frontiers Microbiology)* *1* *. Since all the* *experiments were done at 37ºC, it is not unexpected to find that the stability of IncK2-derivatives is* *lower than that of IncK1-derivatives. Did the authors test if the IncK2 minireplicons at 42ºC have a* *stability comparable to the IncK1 at 37ºC, so that they are not discarded from the work? Did the* *authors tested different temperatures?*

 The authors did not test stability of these plasmids at different temperatures. It is possible that replication and stability of IncK2 plasmid is dependent on the temperature.  An appropriate comment is now added in the 3 ^rd^ paragraph of the discussion of the manuscript.

***Specific concerns:*** ***Introduction*** *.* ***1*** ***st*** ***Paragraph*** *. It states that “there are 40 plasmid types described, mostly based on their* *incompatibility”. There are 40 types according to the incompatibility, but additional classification* *methods are being used (MOB, MPF, PTUs,…) and they not included in these 40 aforementioned* *types. I would change ‘mostly based on’ by ‘according to’.* 

 The change was made according to the reviewer’s suggestion.

***2*** ***nd*** ***Paragraph.*** *It could be convenient a reference to the definition of incompatibility.*

 The appropriate reference for a definition of incompatibility was added.

***3*** ***rd*** ***Paragraph*** *. The authors mention that plasmid replication regulated by antisense RNA occurs* *in IncF, IncL, IncM and I-complex plasmids. However, within the references cited (5-11), there is* *also the plasmid R1162 (reference 9, which is an incQ) and the gram positive plasmid pT181* *(reference 5, typed as rep7a). Additionally, ColE1 replication could be added to the list.*

 The change was made according to the reviewer’s suggestion

***4*** ***th*** ***Paragraph*** *. A schematic figure representing the replication mechanism of I-complex plasmids* *(with the role of RNAI) might be useful for readers not used to this plasmid complex. The second* *half of the **5*** ***th*** ***paragraph*** *could also benefit of the figure. Additionally, it could include the general* *genetic map of the minireplicons used (present in the extended data)*

 The authors agree that a schematic figure would be useful. However, since the I-complex replication mechanism in general is not the main focus of the paper and would take up a relatively large space, a relevant reference with an excellent description has been included.

***Methods*** *.* ***Plasmids and vectors used.*** *Either in the text and/or in the table could be indicated which wt* *plasmid is IncK1 or Inck2 (pT.109 or p754).*

  An appropriate addition was made both to the text and the table.

***Incompatibility testing.*** *The minireplicons were electroporated because they are nonconjugative,* *the authors wrote. But, the full wt plasmids were electroporated as well (next* *sentence). Are they also non-conjugative? What is their size? No information on the manuscript.*

 The wt full plasmids are conjugative, but they were electroporated in order to standardize the methods. An appropriate comment is made in the text.

***Results*** *.* ***RNAI structure comparison*** *. The last sentence of the 1* *st* *paragraph (“The predicted size…* *…interior loop of the molecule”) could fit better in the second paragraph, with the result of* *G25T/T24G, or at least after mentioning that some substitutions were tested.*

 The change was made according to the reviewer’s suggestion.

***Figure 1.*** *The RNAfold server has the option to color the nucleotides according to the base-pair* *probability. Have the authors considered the option to include the probabilities in the secondary* *structure inference? Do the wt RNAI’s secondary structures show high probabilities? And the* *mutated ones?* 

 The colored version of the graph generated by the RNAfold was not used because in the colored version single nucleotides were not visible and it would be difficult to show where the actual point mutations were made. Both wt IncK1 and IncK2 RNAI structures show overall very high base-pair probabilities. Introduced point mutations might slightly reduce base-pair probabilities, but don’t drastically change the whole structure integrity.

***Minireplicon stability.*** *Does the minireplicons’ stability correlate with the one of the wt original* *plasmids? Is pT.109 more stable than p754 in these conditions?*

  Stability of wt IncK1 and IncK2 plasmids was shown to be very high (98-100%) therefore observed stability of the minireplicons is much lower. As suggested by the reviewer, a possible explanation as to why the IncK2 minireplicon is much more unstable compared to the IncK1 minireplicon could be that the IncK2 plasmid replication is adapted to 42 ^o^C and performs semi-optimal at 37 ^o^C. A remark has been added in the discussion (3rd paragraph). 

***Minireplicon stability.*** *Some SNPs in the RNAI of IncK2 increase/decrease the stability, but the* *secondary structure (in Figure 1) is really conserved. Do the base-pair probabilities of these* *structures change?*

 The base-pair probabilities of these structures don’t seem to change drastically. Although the structure is conserved, if these mutations occur, they might indeed affect the interactions of RNAI with its target and therefore altering stability of the whole minireplicon.

***Minireplicon incompatibility*** *. ‘…against the parental and non-parental IncK wt plasmids’ means* *against pT.109 and p754?*

 By “parental and non-parental IncK wt” the authors mean wt IncK plasmids (pT.109 and p754). Parental plasmid for IncK1 minireplicon will be wt IncK1 and non-parental wt IncK2 plasmid.

***Figure 4.*** *For the representation of this Figure, might the authors benefit from a stacked* *histogram.*

 The authors chose for this data representation to be able to show standard deviation between three replicates of the experiment, which would be difficult in a stacked histogram.

***Discussion.*** ***1*** ***st*** ***Paragraph.*** *In addition to the RNAI differences, how similar are the rep genes? Could these* *rep genes also provide some incompatibility? Additionally, how conserved are these RNAI in* *nature? All IncK1 and IncK2 plasmids have the exact same RNAIs or are there differences* *dissimilarities in between wt plasmids?* 

 The rep genes of pT.109 and p754 share little DNA identity. RNAI has a complementary region in *repBA* mRNA. Besides that, the authors are not aware of any involvement of the *repA* gene in the incompatibility. To our best knowledge there is no variation in IncK1 and IncK2 RNAI sequence, which is very conserved.

***2*** ***nd*** ***Paragraph.*** *If full wt plasmids should be more stable (toxin/antitoxin, partitioning), one would* *expect them to be more stable when assessing the compatibility, not being more loss than the* *minireplicon (as it is in Figure 4). Any explanation? Are these full wt plasmids more stable than the* *minireplicons? No data on this aspect in the text.*

 Wt IncK1 and IncK2 plasmids are more stable than their minireplicons. However in a direct competition between them it might play a role which plasmid is resident (wt IncK) and which one is incoming (minireplicon). Moreover, the minireplicon will probably have smaller fitness cost compared to the wt plasmid, based on its size and the number of encoded genes.

***2*** ***nd*** ***Paragraph.*** *“The observed incompatibility is weaker than expected”. What were the* *expectation of the authors?*

  Additionally, the fact that the minireplicons constructed in this study lack the partitioning system, may also contribute to the relatively weak observed incompatibility

***2*** ***nd*** ***Paragraph.*** *The authors state that “the results indicate that replication-based incompatibility* *may not be a straight-forward system based on plasmids being present in one cell.” But indeed,* *this is exactly what is occurring in the experiment. Among the bacteria carrying both the full IncK1* *and the minireplicon IncK1, 0% of the colonies tested carry both plasmids after just one overnight.* *Despite other plasmid characteristics may be involved, it does suggest that incompatibility is* *infeluncing the compatibility via replication-based mechanisms.*

 What the authors meant by this statement was that almost none of shown mutations result in full incompatibility. In the pairs of IncK1 minireplicon and IncK2 plasmid, heteroplasmid cells were detected, but no cells harbored only IncK1 minireplicon. These pairs show lower level of incompatibility then IncK1 wt with an IncK1 minireplicon.

***3*** ***rd*** ***Paragraph*** *. Full stop (“.”) missing between reference 21 and the next sentence (“Preserving the* *structure…”).* 

 The change was made according to the reviewer’s suggestion.

***3*** ***rd*** ***Paragraph*** *. “T10C causes the highest increase in plasmid copy number”. In results, it was* *shown that there are no statistical differences in copy number between the IncK1 minireplicons.*

 The incorrect statement was deleted from the manuscript.

***4*** ***th*** ***Paragraph*** *. Do the authors have references for ‘the most used method to determine* *compatibility of two plasmids is conjugation?’*

 The following reference were added to that statement *. * References: Rozwandowicz M, Brouwer MS, Zomer AL, Bossers A, Harders F, Mevius DJ, Wagenaar JA, Hordijk J. Plasmids of Distinct IncK Lineages Show Compatible Phenotypes. Antimicrob Agents Chemother. 2017 Feb 23;61(3):e01954-16.

***References*** *1. Rozwandowicz M, Brouwer MSM, Mughini-Gras L, Wagenaar JA, et al.: Successful Host* *Adaptation of IncK2 Plasmids.Front Microbiol. 2019; **10**: 2384 PubMed Abstract | Publisher Full Text* ***Is the work clearly and accurately presented and does it cite the current literature?*** *Yes* ***Is the study design appropriate and does the work have academic merit?*** *Yes* ***Are sufficient details of methods and analysis provided to allow replication by others?*** *Yes* ***If applicable, is the statistical analysis and its interpretation appropriate?*** *Yes* ***Are all the source data underlying the results available to ensure full reproducibility?*** *Yes* ***Are the conclusions drawn adequately supported by the results?*** *Yes* ***Competing Interests:*** *No competing interests were disclosed.* ***Reviewer Expertise:*** *Plasmid biologist. Mobile Genetic Elements. Bacterial evolution.* ***I confirm that I have read this submission and believe that I have an appropriate level of*** ***expertise to confirm that it is of an acceptable scientific standard, however I have*** ***significant reservations, as outlined above.***
